# Epigenetic Regulation of Vascular Smooth Muscle Cell Phenotype Switching in Atherosclerotic Artery Remodeling: A Mini-Review

**DOI:** 10.3389/fgene.2021.719456

**Published:** 2021-08-05

**Authors:** Michelle Zurek, Einari Aavik, Rahul Mallick, Seppo Ylä-Herttuala

**Affiliations:** A. I. Virtanen Institute for Molecular Sciences, University of Eastern Finland, Kuopio, Finland

**Keywords:** arterial remodeling, epigenetic modifications, atherosclerosis, vascular smooth muscle cells (VMSCs), phenotype switching

## Abstract

Atherosclerosis is a chronic inflammatory disease characterized by extensive remodeling of medium and large-sized arteries. Inward remodeling (=lumen shrinkage) of the vascular walls is the underlying cause for ischemia in target organs. Therefore, inward remodeling can be considered the predominant feature of atherosclerotic pathology. Outward remodeling (=lumen enlargement) is a physiological response compensating for lumen shrinkage caused by neointimal hyperplasia, but as a pathological response to changes in blood flow, outward remodeling leads to substantial arterial wall thinning. Thinned vascular walls are prone to rupture, and subsequent thrombus formation accounts for the majority of acute cardiovascular events. Pathological remodeling is driven by inflammatory cells which induce vascular smooth muscle cells to switch from quiescent to a proliferative and migratory phenotype. After decades of intensive research, the molecular mechanisms of arterial remodeling are starting to unfold. In this mini-review, we summarize the current knowledge of the epigenetic and transcriptional regulation of vascular smooth muscle cell phenotype switching from the contractile to the synthetic phenotype involved in arterial remodeling and discuss potential therapeutic options.

## Introduction

Atherosclerosis (AS) is a chronic multifactorial disorder of medium and large-sized arteries characterized by inflammation and lipid deposition within the arterial wall, leading to slowly progressive plaque formation (Incalcaterra et al., 2013; Xu et al., 2018). Throughout plaque development, reactive changes in the affected vessel wall known as vascular remodeling occur, in which the flow-limiting potential of the atherosclerotic lesion may be accentuated or attenuated (Falk, 2006). In general, arterial remodeling is orchestrated by various cell types, including endothelial cells, macrophages, and vascular smooth muscle cells (VSMCs) (Wei et al., 2013). Unlike most cell types, VSMCs exhibit phenotypic plasticity, allowing them to switch from a contractile to a proliferative state in response to vessel injury. Therefore, VSMC phenotype switching has a tremendous impact on the vascular remodeling process and thus atherosclerotic lesions (Quintavalle et al., 2011). Despite decades of research focusing on understanding the processes controlling VSMC phenotype switching, the effective molecular mechanisms regulating these critical transitions still have not been precisely clarified (Frismantiene et al., 2018). Accumulating evidence shows that epigenetic mechanisms provide a higher level of transcriptional control leading to phenotype switching in vascular cells followed by arterial remodeling (Findeisen et al., 2013).

## Arterial Remodeling in Atherosclerosis

### Arterial Remodeling

Arterial remodeling reflects the structural and functional adaptation of the atherosclerotic vessel wall to biochemical and biomechanical stimuli triggered by disease (van Varik et al., 2012; Jaminon et al., 2019). In general, two main forms of arterial remodeling can be distinguished, namely outward remodeling responsible for an increase in vessel size, and inward remodeling resulting in a reduction of luminal diameter through vascular wall thickening (Ward et al., 2000). Major features of arterial remodeling comprise intimal hyperplasia, changes in extracellular matrix (ECM) composition, fibrosis, and vascular calcification. These wall changes generally are driven by numerous, highly regulated and interrelated processes and different cell types (Endo et al., 2001; Burke et al., 2002; Brasselet et al., 2005; van Varik et al., 2012; Martínez-Martínez et al., 2014).

### Atherosclerosis

**FIGURE 1:**
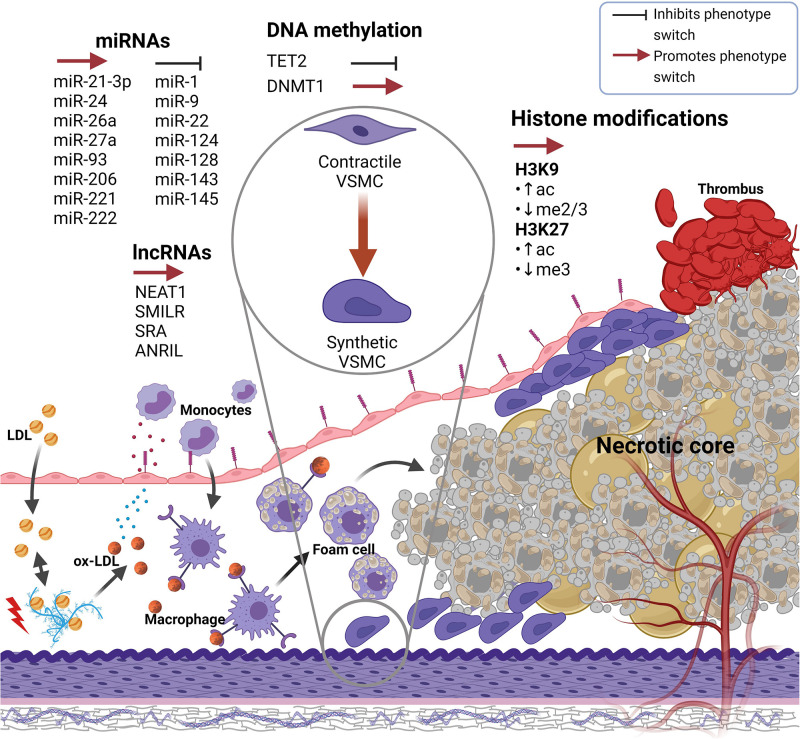
Epigenetic factors either promoting or preventing VSMC phenotype switch in atherosclerotic artery remodeling. Atherosclerosis is initiated when LDL particles accumulate within the subendothelial space where they get oxidized, subsequently causing monocyte extravasation. Monocytes differentiate into macrophages which take up ox-LDL particles and gradually transform into foam cells that undergo cell death and create a necrotic lipid core within the intima. At a specific point in time, plaque rupture and thrombus forming may occur. During atherogenesis, various (epigenetic) factors trigger VSMCs, which reside within the tunica media, to switch from a contractile to a synthetic phenotype. Synthetic VSMCs migrate toward the intima where their proliferate and synthetize extracellular matrix components, resulting in neointima formation and thus arterial remodeling. Created with Biorender.com. VSMC(s), Vascular smooth muscle cell(s); LDL, Low-density lipoprotein; ox-LDL, Oxidized LDL.

In AS, low-density lipoprotein (LDL) particles enter the subendothelial space of the tunica intima, where they get oxidized (i.e., oxLDL) and trigger the activation of endothelial cells (Goikuria et al., 2018). Activated endothelial cells mediate immune cell adhesion and infiltration, eventually leading to the initiation of inflammation (Jaminon et al., 2019). Macrophages in turn scavenge oxLDL and convert into foam cells, thereby building up fatty streaks and producing cytokines that attract activated medial VSMCs to migrate and proliferate into the subendothelial space, ultimately leading to arterial lumen narrowing due to neointima formation (Owens et al., 2004; Douglas and Channon, 2014). The slow advancement of neointimal thickening can lead to ischemia or rupture of the plaque, manifesting in thrombus formation and acute cardiovascular events such as myocardial infarction and stroke (Figure 1; Bentzon et al., 2014).

### Role of Arterial Remodeling in Atherosclerosis

Throughout lesion progression, reactive changes in the underlying vessel wall trigger arterial remodeling processes, in which the flow-limiting potential of the plaque either may be strengthened (i.e., inward remodeling) or attenuated (i.e., outward remodeling) (Falk, 2006). In general, inward remodeling can be regarded as the predominant characteristic of atherogenesis. Even though inward remodeling is correlated with stable atherosclerotic lesions through fibrotic cap formation, it is responsible for more severe luminal narrowing and thus ischemia (Vink et al., 2001). Outward remodeling in atherosclerotic arteries on the other hand prevents ischemia by preserving a normal lumen diameter, but, however, is associated with vulnerable atherosclerotic lesions and thus plaque rupture (Falk, 2006; Saam et al., 2016).

## Vascular Smooth Muscle Cells: the Main Players of Arterial Remodeling

### Vascular Smooth Muscle Cells and Phenotype Switching

Vascular smooth muscle cells play a pivotal role in the different remodeling processes as they are the most abundant cell type found in arterial vessel walls (Lacolley et al., 2017). Mature VSMCs principally are contractile and highly specialized in regulation of vessel tone-diameter and blood pressure (Owens et al., 2004; Rzucidlo et al., 2007), and are characterized by a low proliferation rate and the expression of explicit contractile proteins such as smooth muscle myosin heavy chain 11 (MYH11), calponin, and transgelin (TAGLN) (Owens, 1995).

Lineage-tracing experiments have shown that VSMCs are derived from various distinct progenitor cells in embryogenesis due to which VSMCs typically exhibit lineage-dependent responses to signaling pathways and can have distinct functional characteristics (Basatemur et al., 2019). Besides, these experiments demonstrated that VSMCs give rise to diverse cell types within lesions fulfilling positive as well as negative roles in atherogenesis, which has led to significant improvements in understanding the functional consequences of developmental origin, clonality, plasticity, and fate of VSMCs within atherosclerotic plaques (Basatemur et al., 2019).

However, unlike most cell types, VSMCs are not terminally differentiated and consequently display plasticity in their phenotypes (Babaev et al., 1990), ranging from contractile–quiescent to migratory–proliferative–synthetic and osteogenic, or macrophage-like (Jaminon et al., 2019). Upon vascular disease such as AS, VSMCs transdifferentiate into the synthetic phenotype by downregulation of mature VSMC-specific marker expression (Sobue et al., 1999). Those cells migrate from the media to the intima, where they excessively proliferate and synthesize ECM components and promote lipid deposition, consequently facilitating arterial wall remodeling (Wang et al., 2017). Phenotypically modulated VSMCs within lesions can comprise about 30% of the total cell count as confirmed by lineage tracing experiments (Shankman et al., 2015). When VSMC-derived fibromyocytes are considered to stabilize plaques (Wirka et al., 2019), then activated VSMC transdifferentiating into chondro-/osteoblast or inflammatory cells can lead to plaque destabilization (Miano et al., 2021).

### Factors Regulating VSMC Phenotype Switching and Arterial Remodeling

Alterations in diverse environmental factors modulate the transcriptional regulation of VSMC-specific genes in AS and arterial remodeling (Jaminon et al., 2019; Shi et al., 2019). Main transcription factors involved in VSMC phenotype switch thereby are Krüppel-like factor 4 (KLF4) by activating the pluripotency network of VSMC (Shankman et al., 2015; Jaminon et al., 2019), the master regulator myocardin (MYOCD)—serum response factor (SRF) regulated by, among others, Olfactomedin 2 and Transcription factor 21 (TCF21) (Shi et al., 2019; Nagao et al., 2020), and octamer binding transcription factor (Oct4) promoting plaque stabilization (Alencar et al., 2020; Kansakar et al., 2021).

Collectively, the mechanisms of VSMC phenotype switching and its effect on vascular remodeling are orchestrated by diverse transcription factors, and the mechanisms are still not fully understood (Shi et al., 2019). A large number of VSMC genes are transcriptionally regulated due to mitogenic stimulation, suggesting an upper level of transcriptional regulation that controls gene expression networks rather than individual genes (Zhang et al., 2002). Interestingly, emerging evidence has revealed that VSMCs undergo epigenetic alterations during phenotypic modulation and vascular remodeling, which provide such upper-level regulation of transcription (Shi et al., 2019).

## The Upper-Level Epigenetic Regulators of Vascular Smooth Muscle Cell Phenotype Switching

Epigenetic mechanisms of gene regulation can be defined as transcriptional memory which alter gene expression without changing the genome (Al-Hasani et al., 2019). Overall, three major epigenetic modifications can be distinguished, in particular DNA methylation (Ming et al., 2021), histone modifications (Taylor and Young, 2021), and non-coding RNAs (ncRNAs) (Statello et al., 2021). Importantly, most epigenetic modifications are reversible, but mitotically stable through cell divisions (Almouzni and Cedar, 2016). Epigenetic alterations can be influenced by diverse factors such as environmental stimuli, age, lifestyle and disease state (Alegría-Torres et al., 2011; Mitteldorf, 2015). These alterations, however, modify transcription factor binding and gene expression, which finally impacts the phenotype of a cell remarkably (Liu et al., 2015).

The role of epigenetics in cardiovascular disease is emerging as a critical linker and player at distinct levels, ranging from pathophysiology to treatment (Al-Hasani et al., 2019). Likewise, the importance of epigenetic alterations has been increasingly recognized in AS, vascular remodeling, and VSMC phenotype switching (Alexander and Owens, 2012; Hou and Zhao, 2021). In the following sections, different epigenetic modifications influencing the transition from the contractile to the synthetic VSMC phenotype in arterial remodeling of atherosclerotic lesions will be discussed (Figure 1).

### DNA Methylation

#### Introduction

DNA methylation is mediated by DNA methyltransferases (DNMTs), which, in vertebrates, covalently bind a methyl group predominantly to the cytosine 5′-carbon in the context of a cytidine phosphate guanosine (CpG) dinucleotide (Xu et al., 2018). DNA methylation can be reversed by dilution *via* genome replication without maintenance or inactivation of DNMTs, or by active demethylation facilitated by Ten-eleven translocation (TET) methylcytosine dioxygenases, which catalyze 5-methylcytosine (5-mC) to 5-hydroxymethylcytosine (5-hmC) (Jurkowska et al., 2011). Cytosine hypermethylation of CpG islands within promoter regions can cause chromatin compaction, subsequently resulting in long term transcriptional repression (Bird, 1986; Fisslthaler et al., 2019).

### The Role of DNA Methylation in VSMC Phenotype Switching

Genome-wide investigations concerning the level of DNA methylation in atherosclerotic lesions have documented DNA hypomethylation as a general phenomenon as well as a unique DNA hypermethylation profile, which affects different genes and pathways implicated in AS pathogenesis (Hiltunen et al., 2002; Zaina and Lund, 2014; Aavik et al., 2015). Moreover, research shows that DNA methylation also participates in controlling VSMC phenotype and arterial remodeling (Findeisen et al., 2013). In general, various genes defining VSMC phenotypes have been found to be regulated by DNA methylation, including SRF, platelet-derived growth factor B (PDGF-B) of the endothelial cell GATA-6-PDGF-B pathway, and TAGLN (Montes de Oca et al., 2010).

For example, a study executed by Liu et al., demonstrated that TET2 acts as a master epigenetic regulator of the VSMC phenotype (Liu et al., 2013). In these studies, Liu et al., discovered that knockdown of TET2 inhibits the expression of critical VSMC genes such as MYOCD and SRF, with simultaneous transcriptional upregulation of KLF4, promoting the reactivation of the pluripotency network and thus phenotype switch. On the other hand, overexpression of TET2 provides a contractile VSMC phenotype, restores the 5-hmC epigenetic landscape, and significantly attenuates intimal hyperplasia *in vivo* (Liu et al., 2013). Next to the studies of Liu et al., experiments of Zhuang et al., indicate that the VSMC phenotype and vascular remodeling are influenced by the DNA methylation balance controlled by TET2 and DNMT1. Decreased expression of TET2 attributes to disproportionate promoter methylation, while inhibition of DNMT1 caused the enrichment of 5-hmC in the MYOCD promoter and prevented VSMC dedifferentiation, migration, and proliferation (Zhuang et al., 2017).

### Histone Modifications

#### Introduction

Histones are the central protein components of chromatin (Shechter et al., 2007). In general, histone proteins can carry various post-translational modifications (PTMs) on their N-terminal tail region, which play a critical role in several DNA-based processes including chromatin accessibility, nucleosome dynamics, and transcription (Lawrence et al., 2016). For this reason, they serve as epigenetic indicators of chromatin state associated with gene activity (Kimura, 2013).

Histone PTMs include, among others, methylation, acetylation, and ubiquitinylation, which principally can be found on arginine (R) and lysine (K) residues (Lawrence et al., 2016). Typically, these modifications can be observed to exist in combinations, such as di- (me2) or tri-methylation (me3) at histone H3K4 together with H3K9ac or H3K14ac, which all have an activating effect on gene expression (Kimura, 2013). Overall, acetylation by histone acetyltransferases (HATs) weakens the interaction between DNA and histone, making genes more accessible for transcription. The removal of an acetyl group by histone deacetylases (HDACs), on the contrary, strengthens the binding between DNA and histone, resulting in repression of gene expression (Gomez et al., 2015; Jiang et al., 2018).

### The Role of Histone Modifications in VSMC Phenotype Switching

Different histone methylation and acetylation changes have been identified to serve a crucial role in the development of AS and VSMC differentiation toward the synthetic phenotype, including significant decrease in H3K9 and H3K27 methylation (Greißel et al., 2015) with concomitant increase in H3K9 and H3K27 acetylation in advanced atherosclerotic plaques (Greißel et al., 2016). The role of H3K27 methylation in VSMC phenotype switching has also been observed in experiments of Wierda et al., who found that a reduction in H3K27me3 in cells of the tunica media plays a significant role in the differentiation and proliferation of VSMCs in AS (Wierda et al., 2015). A study of Harman et al. (2019) showed that reduced H3K9me2 levels in VSMC within atherosclerotic plaques and arteries undergoing injury-induced remodeling is connected to augmented transcription at inflammation-responsive genes. Next to H3K27 and H3K9, H3K4 methylation changes have been correlated with stage-specific progression of AS (Jiang et al., 2018).

Experiments of Chen et al. (2017) demonstrated that increased expression of the histone demethylase KDM3a in diabetic rats promotes neointimal hyperplasia through a reduction in H3K9 di-methylation at the ROCK2 and AGTR1 loci, indicating that the switch from the contractile to the synthetic VSMC phenotype is enhanced by the activation of the Rho/ROCK and AngII/AGTR1 pathways. Moreover, studies of McDonald and Yoshida et al., demonstrated that PDGF B-induced phenotype switching of cultured VSMCs decreases histone acetylation mediated by Klf4-dependent recruitment of HDACs 2, 4, and 5 to various CArG dependent VSMC marker genes and enrichment of the silencing modification H3K9me3 on VSMC promoters (McDonald et al., 2006; Yoshida et al., 2007).

### ncRNAs

Recent discoveries in molecular biology have revealed that gene expression is primarily regulated not only by proteins but also by ncRNAs. Among those, microRNAs (miRNAs) and long non-coding RNAs (lncRNAs) play a crucial role in the transcriptional regulation of genes at diverse levels (Panni et al., 2020; Statello et al., 2021). To date, a large number of distinct ncRNAs has been associated with the VSMC phenotype transition from the contractile to the synthetic phenotype.

### MicroRNA Involvement in VSMC Phenotype Switching

MiRNAs are small RNA molecules with an average length of 22 nucleotides that typically regulate mRNA expression negatively by binding to a complementary sequence often located in the 3′ UTR region of the target mRNA (Ambros, 2004; Zhang et al., 2017). Table 1 summarizes recently discovered as well as extensively studied miRNAs affecting VSMC phenotype switch from the contractile to the synthetic VSMC phenotype in a direct or indirect way.

**TABLE 1:** Recently discovered and well-studied miRNAs affecting VSMC phenotype switch from the contractile to the synthetic phenotype in vascular remodeling of atherosclerotic arteries.

miRNA	Promoted VSMC phenotype	Target mRNA(s)	References
miR-1	Contractile	KLF4, Pim-1, and HDAC4	Chen et al., 2011; Xie et al., 2011
miR-9	Contractile	PDGFR	Ham et al., 2017
miR-21-3p	Synthetic	PTEN	Zhu et al., 2019
miR-22	Contractile	MECP2, EVI1, and HDAC4	Yang et al., 2018
miR-24	Synthetic	Trb3	Yoshida et al., 2013; Cai et al., 2019
miR-26a	Synthetic	Smad-1	Yang et al., 2017
mIR-27a	Synthetic	α-SMA	Xu et al., 2019
miR-30b-5p	*Unidentified*	MBNL1	Woo et al., 2019
miR-93	Synthetic	Mfn2	Feng et al., 2019
miR-124	Contractile	Sp1	Tang et al., 2017
miR-128	Contractile	KLF4, MYH11	Farina, Hall et al., 2020
miR-143, miR-145	Contractile	KLF4, Elk-1, ACE, and UHRF1	Boettger et al., 2009; Cordes et al., 2009; Elia et al., 2018
miR-206	Synthetic	ZFP580	Sun et al., 2017
miR-221	Synthetic	P27kip1, c-kit	Liu et al., 2009
miR-222	Synthetic	P27kip1, P57kip2	Liu et al., 2009

### The Role of Long Non-coding RNAs in VSMC Phenotype Switching

LncRNAs are commonly defined as non-protein-coding transcripts larger than 200 nucleotides (Boon et al., 2016). Through interaction with DNA, RNA, and proteins, lncRNAs can affect chromatin function and structure, the transcription of neighboring and distant genes, and modulate RNA translation (Josefs and Boon, 2020; Statello et al., 2021). In the following, recently identified lncRNAs will be discussed.

Interestingly, the most significant cardiovascular disease related genomic locus Chr9p21.3 includes ANRIL lncRNA, which promotes VSMC phenotype switching when overexpressed through possibly acting as a molecular scaffold to promote WDR5 and HDAC3 complex forming (Zhang et al., 2020). LncRNA nuclear paraspeckle assembly transcript 1 (NEAT1), on the other hand, seems to promote VSMC phenotype switch from a contractile to a synthetic cell type by inhibiting the chromatin modifier WDR5, normally stimulating the expression of contractile VSMC specific genes (Ahmed et al., 2018). Another lncRNA, being SMILR (smooth muscle–induced lncRNA), directly binds mitotic protein centromere protein F (CENPF) mRNA and promotes VSMC proliferation and cell cycle progression (Mahmoud et al., 2019). In agreement with VSMC proliferation conferring with atherosclerotic plaque stability, increased SMILR levels have been detected in unstable compared with stable human lesions in an investigation of Ballantyne et al., 2016 (Ballantyne et al., 2016). Another lncRNA influencing VSMC proliferation and thus arterial remodeling is steroid receptor RNA activator (SRA), which generally modulates common proliferative kinase pathways. SRA overexpression experiments in a wire-injury murine model of vascular injury for example tremendously increased neointimal hyperplasia by influencing the MEK-ERK-CREB pathway (Zhang et al., 2019).

## Epigenetic Therapeutic Options

As can be seen, various epigenetic modifications can either promote or prevent arterial remodeling in AS through directly and indirectly affecting the phenotype of VSMCs. In recent years, several target genes and molecules regulating all classes of epigenetic modifications have been examined in animal models and clinical trials for their effects in the general treatment of AS (Jiang et al., 2018). The DNMT inhibitor 5-aza-2′-deoxycytidine (5-aza-dC) for instance has shown to prevent the depressed expression of methylated genes and is at present approved for the treatment of myelodysplastic syndrome (Mossman et al., 2010). 5-aza-dC *in vitro* experiments by Zhuang et al., decreased global 5-mC content and re-established myocardin expression in VSMCs induced by PDGF, thus inhibiting excessive VSMCs dedifferentiation (Zhuang et al., 2017). However, 5-aza-dC does not work target specific manner and can induce pluripotency together with a risk for developing cancer (Taylor and Jones, 1979). Another category of chemical compounds influencing epigenetic modifications are HDAC inhibitors (HDACi) such as Vorinostat, which currently are clinically utilized as anticancer agents (Suraweera et al., 2018). A study of Ye et al., showed that Vorinostat generally reduced atherosclerotic plaque size in ApoE^–/–^ mice. In this experiment, differentially expressed mRNAs and ncRNAs as well as their interactions and pathways were identified, which to some extent explain the anti-atherosclerotic effect of this HDACi (Ye et al., 2018). The effects of Vorinostat on VSMC specific epigenetic modifications could be investigated in follow up experiments. On the other hand, miRNA-based therapeutics, which currently are in preclinical development, work with synthetically derived oligonucleotide duplexes mimicking target specific miRNAs or work as antisense-miRNAs. Next to cancer, *in vivo* delivery of miRNA-based therapeutics has been successfully performed in murine models of cardiac diseases, hepatitis, and diabetes-associated kidney fibrosis, making it an interesting approach in modulating VSMC phenotype switching (Saliminejad et al., 2019).

## Discussion and Future Perspectives

In conclusion, current research increasingly reveals the critical role of epigenetic transcriptional regulation in VSMC phenotype switching. Nevertheless, a large number of factors and mechanisms still need to be clarified. AS is a multifactorial disease, and arterial remodeling and plaque composition present a high level of interindividual heterogeneity (Box et al., 2007; Incalcaterra et al., 2013). Epigenetic modulations also are typically controlled by multiple factors like environmental parameters, age, lifestyle, and the gut microbiome, making the identification of factors and pathways initiating and controlling the epigenetics of VSMC phenotype switching a difficult task (Alegría-Torres et al., 2011; Mitteldorf, 2015; Alam et al., 2017).

Regarding future epigenetic therapeutical options, antisense oligonucleotide therapy (AOT) seems to present a promising approach to VSMC phenotype modulation due to its target specificity. At present, antisense oligonucleotides against lipoprotein small a (Ionis Pharmaceuticals, CA, United States) appear to form an attractive way for the treatment of AS (Tsimikas et al., 2020). Extracellular vesicles loaded with proteins, DNA oligonucleotides or RNA, on the other hand, form a potential theoretical approach to fight against trained immunity (i.e., prolonged secretion of inflammatory cytokines), continuously triggering arterial remodeling and thus VSMC phenotype switch (Doyle and Wang, 2019). However, for this purpose, the epigenetic modifications responsible for trained immunity, as well as the possibilities to enhance trained immune tolerance in patients, need to be elucidated.

## Author Contributions

SY-H devised the topic and the principal conceptual ideas. MZ and EA mainly wrote the manuscript. RM contributed to the design of the figure. All authors discussed and commented on the manuscript.

## Conflict of Interest

The authors declare that the research was conducted in the absence of any commercial or financial relationships that could be construed as a potential conflict of interest.

## Publisher’s Note

All claims expressed in this article are solely those of the authors and do not necessarily represent those of their affiliated organizations, or those of the publisher, the editors and the reviewers. Any product that may be evaluated in this article, or claim that may be made by its manufacturer, is not guaranteed or endorsed by the publisher.
